# Comparison of Plasma Metabolome Response to Diets Enriched in Soybean and Partially-Hydrogenated Soybean Oil in Moderately Hypercholesterolemic Adults-A Pilot Study

**DOI:** 10.3390/metabo13040474

**Published:** 2023-03-26

**Authors:** Neil K. Huang, Alice H. Lichtenstein, Gregory Matuszek, Nirupa R. Matthan

**Affiliations:** 1 Cardiovascular Nutrition Laboratory, Jean Mayer USDA Human Nutrition Research Center on Aging at Tufts University, Boston, MA 02111, USA; 2 Bionformatics Core Unit, Jean Mayer USDA Human Nutrition Research Center on Aging at Tufts University, Boston, MA 02111, USA

**Keywords:** partially-hydrogenated fat, *trans* fatty acids, soybean oil, randomized controlled crossover feeding trial, metabolites

## Abstract

Partially-hydrogenated fat/*trans* fatty acid intake has been associated with adverse effects on cardiometabolic risk factors. Comparatively unexplored is the effect of unmodified oil relative to partially-hydrogenated fat on the plasma metabolite profile and lipid-related pathways. To address this gap, we conducted secondary analyses using a subset of samples randomly selected from a controlled dietary intervention trial involving moderately hypercholesterolemic individuals. Participants (N = 10, 63 ± 8 y, BMI, 26.2 ± 4.2 kg/m^2^, LDL-C, 3.9 ± 0.5 mmol/L) were provided with diets enriched in soybean oil (SO) and partially-hydrogenated soybean oil (PHSO). Plasma metabolite concentrations were determined using an untargeted approach and pathway analysis using LIPIDMAPS. Data were assessed using a volcano plot, receiver operating characteristics curve, partial least square-discrimination analysis and Pearson correlations. Among the known metabolites higher in plasma after the PHSO diet than the SO diet, the majority were phospholipids (53%) and di- and triglycerides (DG/TG, 34%). Pathway analysis indicated upregulation of phosphatidylcholine synthesis from DG and phosphatidylethanolamine. We identified seven metabolites (TG_56:9, TG_54:8, TG_54:7, TG_54:6, TG_48:5, DG_36:5 and benproperine) as potential biomarkers for PHSO intake. These data indicate that TG-related metabolites were the most affected lipid species, and glycerophospholipid biosynthesis was the most active pathway in response to PHSO compared to SO intake.

## 1. Introduction

Diet quality, especially dietary fat type, plays a crucial role in modulating cardiometabolic risk factors (CMRF) and, subsequently, cardiovascular disease (CVD) risk [1,2]. As a modifiable behavior, it is a first-line approach for CVD risk reduction. Replacing saturated fatty acids (SFA) with polyunsaturated (PUFA) and monounsaturated fatty acids (MUFA) has been demonstrated to lower the incidence of CVD [2,3,4,5,6,7,8,9], with the exception of one specific group of dietary unsaturated fatty acids, *trans* fatty acids (TFA). These fatty acids are produced as a result of biohydrogenation in the ruminant fat of animals (rTFA) or by industrial hydrogenation (iTFA) of plant oils [10]. Soybean oil is the most widely produced and consumed edible oil in the U.S. [11]. Prior to 2018, the majority of TFAs in the U.S. diet were contributed by products made with partially-hydrogenated oils, such as commercially baked goods, fried foods and margarines [12]. Evidence from clinical trials and prospective observational studies consistently document the adverse effects of TFAs on plasma LDL-cholesterol concentrations and coronary heart disease (CHD) morbidity and mortality, and all-cause mortality [13,14]. Based on available evidence, the World Health Organization has called for the elimination of iTFAs from the global food supply by 2023. In 2018 the US removed partially-hydrogenated fat from the Food and Drug Administration’s (FDA) Generally Recognized as Safe list. However, iTFAs are still prevalent in foods available in countries that have not enacted similar policies. Thus, there is still a need to further document and understand the mechanisms behind the adverse effects of TFAs.

Metabolomics is a promising approach to objectively assess the impact of diet on chronic disease risk by quantifying small molecules in a biological system [1,15]. The majority of published literature using untargeted metabolomics in nutrition research has been biomarker discovery based on individual foods and dietary patterns [15,16,17,18,19,20]. Fewer studies have compared metabolomic profiles among dietary fats and oils [21], especially compared metabolite profiling between soybean oil and partially-hydrogenated fat [22,23] or determined the association with underlying metabolic pathways.

We have previously reported that consuming partially-hydrogenated soybean oil (PHSO) relative to unmodified (native) soybean oil (SO) as well as SO varieties with modified fatty acid profiles resulted in an unfavorable lipid profile in moderately hypercholesterolemic older women and men [24]. Using a subset of samples from this randomized crossover controlled feeding trial, the aim of this exploratory trial is to use an untargeted metabolomics approach to comprehensively evaluate plasma metabolite profiles, and associated pathways, after participants consume diets enriched with two different forms of soybean oil, unmodified SO and PHSO containing margarine. Our hypotheses are that SO and PHSO would result in distinct plasma metabolite profiles and these profiles would be differentially associated with lipid-related metabolic pathways.

## 2. Methods and Materials

### 2.1. Study Participants, Design and Diet Intervention

This exploratory study was a secondary analysis using plasma samples collected from a subset of 10 subjects who participated in a randomized controlled crossover feeding trial evaluating the effect of novel soybean oils with differing fatty acid profiles on CVD risk factors in moderately hyperlipidemic subjects [24]. The subjects were randomly selected from the original cohort. A detailed description of the parent study design, screening and recruitment criteria, diet composition and results, including serum lipid and lipoprotein concentrations, was reported previously [24]. Briefly, moderately hypercholesterolemic men and postmenopausal women (≥50 years with LDL-C concentration ≥3.36 mmol/L.) were recruited from the greater Boston area. Participants consumed each diet randomly for 5 weeks, with a 2-week washout period between diet phases. Prior work has documented that a 2-week washout period between diet phases is sufficient to allow no carryover or order effects [25,26]. The participants, investigators, and all laboratory staff were blinded to the order of the diet phases [24]. The trial that provided samples for the present study was registered at clinicaltrials.gov as NCT00175071.

Diets were designed to provide similar amounts of carbohydrate (~53% energy), protein (~17% energy), and fat (30% energy) (Supplementary Table S1), with two-thirds of the fat (20% energy) contributed by the experimental oil or fat. Participants only consumed the food provided without additional foods and beverages, except water. The diet composition was analyzed by chemical analyses (Covance Laboratories Inc., Madison, WI, USA). Study menus have appeared previously [24]. For this study, we focused on two diet phases, unmodified (native) soybean oil (SO, Solae Company, (St Louis, MO, USA) and partially hydrogenated soybean oil (PHSO) from a commercially available margarine product (Whirl; Proctor and Gamble Company, Cincinnati, OH, USA). The PHSO-enriched diet provided approximately 3% of energy as TFAs. The baseline characteristics of the 10 participants with an archived blood sample (unthawed and stored at −80 °C) available from both SO and PHSO phases for the present untargeted metabolomics analysis are presented in Table 1. The parent study, registered at clinicaltrials.gov as NCT00175071, was conducted between 2001 to 2005 in accordance with the Declaration of Helsinki guidelines. All procedures were approved by the Institutional Review Board of Tufts University/Tufts Medical Center.

### 2.2. Untargeted Metabolomics

Fasting plasma metabolite profiles were determined using an untargeted metabolomics approach by either gas chromatography/quadrupole time-of-flight tandem mass spectrometry (GCTOF MS for primary metabolism-related metabolites [carbohydrates and sugar phosphates, amino acids, hydroxyl acids, free fatty acids, purines, pyrimidines, aromatics, exposome-derived chemicals]); BEH C18-quadrupole time-of-flight tandem (QTOF MS/MS for complex lipids [ceramides, sphingomyelins, cholesteryl esters, lyso- and phospholipids]) and ultra-high pressure liquid chromatography/quadrupole time-of-flight tandem mass spectrometry (UHPLC-QTOF MS/MS for biogenic amines [acylcarnitines, TMAO, cholines, betaines, SAM, SAH, nucleotides and nucleosides, methylated and acetylated amines, di- and oligopeptides) methodology at the University of California, Davis (West Coast Metabolomics Center). Data quality was ensured by including pooled and blank samples, and automatic liner exchanges minimized sample carryover for highly lipophilic compounds.

#### 2.2.1. Primary Metabolites, Complex Lipids and Biogenic Amines Extraction, Data Acquisition and Processing

The sample preparation, method and data processing for GCTOF MS have been described previously [27,28,29]. The signal/noise levels of the chromatogram for peak detection and automatic mass spectral deconvolution were set at 5:1, and the mass-to-charge ratio was reported for use in the BinBase algorithm. The peak alignment and metabolite detection for lipids were processed using Mass Hunter qualitative analysis, Mass Profiler Professional and Mass Hunter quantitative analysis (Agilent Technology, Santa Clara, CA, USA). Lipidblast was chosen for library matching [30]. The free mzMine 2.0 software [31], NIST14/Metlin/MassBank online libraries, and Mass Hunter quantitative analysis (Santa Clara, CA, USA) were utilized for biogenic amines data analysis.

#### 2.2.2. Pathway Analysis

Lipid pathway analysis was analyzed and visualized using Lipid Maps^®^ (https://www.lipidmaps.org/, accessed on 10 December 2022) [32] to identify pathways of relevance and potential physiological functions using metabolites that exhibited significant statistical differences and distinguished the diet groups.

### 2.3. Plasma Lipid and Phospholipid Fatty Acid Profiles

TC, TG and high-density lipoprotein-cholesterol (HDL-C) concentrations were measured as previously described [24] using a Hitachi 911 automated analyzer (Ingelheim, Germany) with enzymatic reagents (coefficient of variation <3%). The Friedewald equation was employed to calculate LDL-C concentrations [33]. The VLDL-C concentrations were estimated by the following formula: TC − (LDL-C + HDL-C). For phospholipid fatty acid analysis, the phospholipid subfraction in plasma was separated by solid-phase extraction using aminopropyl columns, the fatty acids were methylated, and the resulting fatty acid methyl esters (FAMEs) were analyzed on a gas chromatograph equipped with a flame ionizing detector, using an established method as previously reported [24,34].

### 2.4. Statistical Analysis

Participant characteristics are presented as mean ± standard deviations (S.D.) for continuous measures and proportions for categorical measures. The lipid and lipoprotein profiles at the end of PHSO and SO diet phases were compared using the paired *t*-test (SAS for Windows Version 9.4; SAS Institute, Cary, NC, USA), with additional adjustments for confounding factors, including diet phase, sequence, age, body mass index and sex.

For metabolomics profiling, a total of 3028 metabolites were measured, of which 841 were identified. Data were log-transformed, normalized, and filtered by the interquartile range for the 841 known metabolites prior to data analysis. When the minimum detectable values (less than 80%) were imputed, 12 known metabolites were excluded (*n* = 829).

Volcano plots (MetaboAnalyst, version 5.0) [35] were used to analyze and visualize the data, and the Benjamini & Hochberg procedure was employed to adjust for multiple comparisons (0.05/829, *p* < 6.03 × 10^−5^; FDR < 0.05). Based on the results from the volcano plot analysis, the seven metabolites were identified with VIP scores >1.7, calculated using partial least squares discriminant analysis (PLS-DA). A full list of identified metabolites ranked from the highest to the lowest after using a cutoff of 1.7 is provided in Supplementary Table S2. ROC curves were computed to determine the accuracy of the potential biomarkers. The areas under the curves and the confidence intervals were determined using a cutoff of 0.7 as a threshold to select metabolites. The Z score was calculated to identify the most active and suppressed lipid-related pathways between two diet phases (cutoff: 1.96 for significance) in Lipid Maps^®^.

Pearson correlation coefficients were computed to assess the relationships between diet-derived metabolites (*n* = 7) and LDL-cholesterol, VLDL-cholesterol and HDL-cholesterol concentrations using R (version 3.6.3; Vienna, Austria). Statistical significance was defined as two-tailed α ≤ 0.05.

## 3. Results

### 3.1. Participant Characteristics and Serum Lipid and Phospholipid Fatty Acid Profiles after Consumption of SO and PHSO-Enriched Diets

The mean age ±S.D. of the subset of participants included in this study (N = 10; 4 postmenopausal women and six men) was 63 ± 8 y, and their BMI was in the overweight category (26.2 ± 4.2 kg/m^2^). At baseline, the participants had elevated LDL-cholesterol concentrations (3.9 ± 0.5 mmol/L). Total cholesterol, HDL-C and triglyceride (TG) concentrations were 5.8 ± 0.8 mmol/L, 1.3 ± 0.3 mmol/L and 1.6 ± 0.7 mmol/L, respectively. Consumption of the PHSO, compared with SO, for five weeks significantly increased total cholesterol and LDL-C concentrations (Table 1). No significant diet effects were observed in other lipoprotein or TG concentrations. The magnitude of change in LDL-cholesterol was similar to that previously reported for the original cohort, confirming that this subset is representative of the larger cohort with regard to lipoprotein responses.

**Figure 1:**
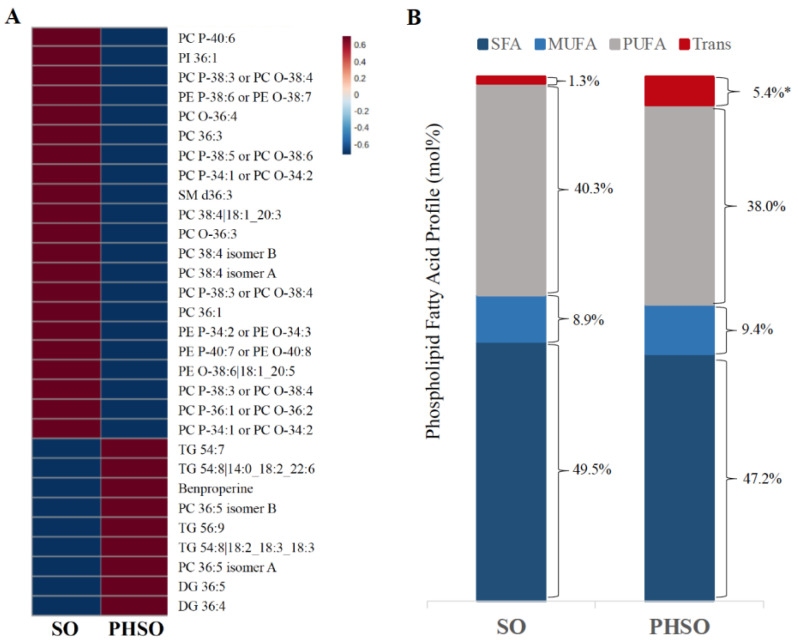
Plasma metabolite (**A**) and phospholipid fatty acid (**B**) Profiles after participants consumed the soybean oil (SO) and partially-hydrogenated soybean oil (PHSO) enriched diets. *, *p* < 0.01.

Consumption of the PHSO, compared with the SO-enriched diet, resulted in a shift in the plasma concentrations of several lipid related metabolites (Figure 1A), primarily increases in phospholipids and decreases in DG and TG species. Fatty acid profiling of this phospholipid fraction in plasma (Figure 1B) revealed a significantly higher proportion of total *trans* fatty acids after subjects consumed the PHSO than the SO (5.4 versus 1.3 mol %) diet. This was primarily due to the higher incorporation of *trans* 18:1n-9 (elaidic acid), with a smaller contribution from *trans* 18:2 and *trans* 18:3 isomers. There was also a small increase in the MUFA content of *cis* isomers. The relative proportion of total SFAs (50% versus 47%) and PUFAs (40 vs. 38%) was similar to the SO diet after the PHSO.

### 3.2. Metabolite Profiling

Results of a volcano plot indicate that plasma concentrations for triglyceride (TG) 56:9, TG 54:8 (TG 18:2_18:3_18:3), benproperine, diglyceride (DG) 36:5, TG 48:5, TG 54:7 and TG 54:6 (TG 18:2_18:2_18:2) were all significantly higher (all *p* < 0.01) after participants consumed the PHSO-enriched diet compared with the SO-enriched diet (Table 2). The ROC curves for these seven metabolites ranged from 0.78 to 1. PLS-DA revealed that the first and second components explained 7.2% and 12.2% of the variance between SO- and PHSO-enriched diets, respectively. A total of 53 metabolites had VIP scores greater than 1.7 (cutoff threshold), of which 28 were phospholipids, 16 were TGs, 3 were DGs, 3 were xenobiotics, 2 were amino acids, and one was a fatty acid (Table 2). A full list of metabolites is listed in Supplementary Table S2.

### 3.3. Pathway Analysis in Lipid Maps

**Figure 2:**
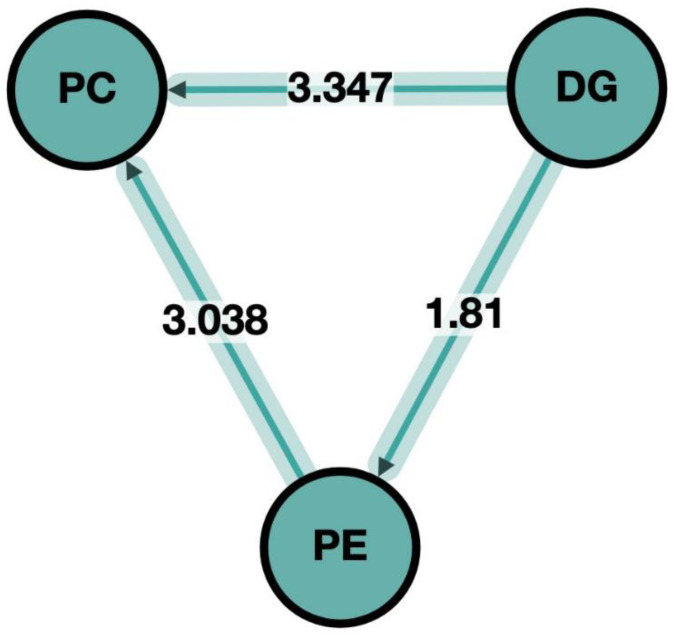
Lipid pathway analysis after participants consumed the soybean oil (SO) and partially-hydrogenated soybean oil (PHSO) enriched diets. The biosynthesis of phosphatidylcholine (PC) from DG and phosphatidylethanolamine (PE) was upregulated after participants consumed the PHSO-enriched diet rather than the SO-enriched diet. Z scores over three were considered significant pathways.

Lipid-related pathway analysis revealed that the most upregulated pathway (Z scores over 3; cutoff: 1.96) after participants consumed the PHSO diet rather than the SO-enriched diet was related to glycolipid and glycerophospholipid synthesis, specifically the biosynthesis of phosphatidylcholine (PC) from DG and phosphatidylethanolamine (PE) (Figure 2). Four associated fatty acid (FA) pathways were upregulated, including: FA(18:1)-FA(20:1)-FA(22:1); FA(16:0)-FA(16:1); FA(18:3)-FA(20:3) and FA(20:2)-FA(20:3).

### 3.4. Associations between Plasma Metabolites and Lipoprotein Fractions

There were three positive associations between plasma metabolites and VLDL and LDL concentrations and no significant associations with HDL concentrations (Table 3). Specifically, TG 56:9 was positively associated with VLDL-cholesterol (r = 0.6, *p* = 0.047) after consumption of the SO diet, but positively associated with LDL-cholesterol after consumption of the PHSO-enriched diet. A significant association was also observed between benproperine and VLDL-C concentrations after consumption of the SO diet (r = 0.77, *p* = 0.009).

## 4. Discussion

This pilot study used an untargeted metabolomics approach to comprehensively assess changes in plasma metabolome and associated lipid-related pathways following consumption of diets enriched in PHSO compared with unmodified SO, identifying potential biomarkers of PHSO and their association with plasma lipoprotein concentrations. Results indicate that the majority of metabolites that distinguished PHSO from the SO diet were TG-related species. These included TG 56:9, TG 54:8 (TG 18:2_18:3_18:3), TG 48:5, TG 54:7 and TG 54:6 (TG 18:2_18:2_18:2), and DG 36:5. Hence, these may be potential biomarkers for diets high in PHSO. Phosphatidylcholine biosynthesis from DG and PE was the major lipid-related pathway altered by PHSO intake.

Few studies have assessed metabolomic profiles in response to dietary TFA or partially hydrogenated fat. Guggisberg et al. applied a combination of targeted and untargeted metabolomics to serum samples from subjects who consumed margarine (iTFAs) or butter (rTFAs) and identified distinct TFA signatures associated with the different dietary fats [23]. The most abundant TFA in butter was C18:1 *t*11 (trans vaccenic acid); in margarine, it was C18:1 *t*9 (elaidic acid). However, no other metabolites that distinguished the two diets were identified, possibly due to the low dose of TFAs (2% of energy intake) and the lack of clinically significant differences in lipid profiles. Of note, *trans* 18:1 (elaidic acid) is the major isomer formed as a result of partial hydrogenation of native oils and has previously been identified as a putative dietary intake biomarker of margarine and hardened vegetable fats, as it cannot be synthesized endogenously by humans [35,36].

Comparing a relatively high intake of *trans* 18:1 (7% of energy) to a diet high in oleic acid for 16 weeks, Gürdeniz et al. documented elevated LDL-cholesterol levels, accompanied by alterations in phospholipid metabolism in overweight postmenopausal women [22]. Lipidomics analysis identified elevated levels of specific PUFA long-chain phosphatidylcholines (PCs) and sphingomyelin (SM). Similarly, Meikle et al. [37] documented significant increases in postprandial phospholipid metabolism after the consumption of dairy fat containing rTFA, compared with SO-enriched meals.

In our study, the metabolomic analysis revealed that phospholipids and TG species were the major metabolites in plasma that were significantly different after participants consumed the PHSO compared to the SO-enriched diet. Fatty acid profiling of the plasma phospholipid fraction confirmed a significantly higher proportion of total TFA (predominantly *trans* 18:1n-9) with PHSO consumption. Among the TG-related metabolites that differentiated the PHSO from the SO-enriched diet, one metabolite, TG 56:9, was significantly associated with plasma LDL-cholesterol concentrations. Interestingly this metabolite was associated with the VLDL-cholesterol concentrations after subjects consumed the unmodified SO diet. Since TGs are major components of VLDL and chylomicrons, this suggests a potential PHSO-induced shift in TG enrichment from VLDL to LDL. Phosphatidylcholine and phosphatidylethanolamine are major phospholipids in mammalian membranes. In the liver, PC is synthesized via the choline pathway or methylation of PE via the enzyme phosphatidylethanolamine N-methyltransferase. Altering the PC/PE ratio has been shown to be a key regulator of cell membrane integrity. Furthermore, in unmodified plant oils, the monoacylglycerols in the sn-2 position are predominantly PUFA or MUFA and serve as primary backbones for gut and liver phospholipid synthesis. Displacing PUFA or MUFA from the critical sn-2 position by substitution with TFA or SFA has been suggested to adversely affect lipoprotein metabolism through the down-regulation of the LDL receptor [38]. Additionally, phospholipids containing TFA behave similarly to SFA rather than to their *cis* MUFA isomers, as TFA-containing acyl chains have similar configurations to SFA-containing acyl chains [39]. These effects could potentially contribute to modulating lipoprotein metabolism [40] and partly explain the increase in LDL-cholesterol concentrations in response to substituting PHSO for SO.

Of note, after consuming the PHSO rather than the SO-enriched diet, participants had higher plasma concentrations of benproperine, which were significantly associated with VLDL-cholesterol. Benproperine is a bioactive molecule that has anti-neoplastic properties [41]. The significance of this finding in the present study is unclear. Given the dramatic decrease in TFA intake resulting from the removal of partially-hydrogenated fat from the U.S. food supply [12], retrospective studies will be necessary to verify this observation.

A strength of this study is the randomized crossover-controlled design which minimized the potential introduction of inter-individual variability among participants. All foods and beverages were provided to the participants, and the compliance rate was high. An untargeted metabolomics approach was applied to this study, which covered a wide range of metabolites. However, only the identified metabolites in this study were included for data analyses, and there is a possibility that unidentified metabolites may also differentiate between SO- and PHSO-enriched diets. Studies with larger sample sizes are necessary to confirm these observations. Additionally, our approach did not allow us to distinguish the proportion of *cis* and *trans* isomers within TG species, so we could not confirm if TFA exists in TG 56:9 and their subsequent involvement with LDL-C concentrations.

## 5. Conclusions

Compared with dietary SO, PHSO intake modified several lipid-related metabolites and upregulated the PC biosynthesis pathway. The most sensitive metabolite was TG 56:9, which was positively associated with LDL-cholesterol after participants consumed the PHSO-enriched diets. The modulation of PC and TG-related pathways and metabolites provides an additional explanation for the positive association between partially-hydrogenated fat intake and cardiovascular disease.

## Figures and Tables

**Table 1:** Cardiometabolic risk factors of the study participants after consumption of the SO and PHSO-enriched diets.

Variables	SO-Enriched Diet	PHSO-Enriched Diet
Total cholesterol, mmol/L	5.3 ± 0.77	5.6 ± 0.76 *
LDL-C, mmol/L	3.4 ± 0.53	3.6 ± 0.55 *
HDL-C, mmol/L	1.3 ± 0.26	1.4 ± 0.29
VLDL-C, mmol/L	0.62 ± 0.34	0.63 ± 0.36
TG, mmol/L	1.7 ± 0.97	1.7 ± 0.86
TC/HDL-C	4.12	4.22
LDL-C/HDL-C	2.62	2.72

Values are presented as mean ± SD (N = 10). The diet effect was assessed using paired *t*-test (*p* < 0.05). HDL-C, high-density lipoprotein-cholesterol; LDL-C, low-density lipoprotein-cholesterol; TC/HDL-C, the ratio of total cholesterol: high-density lipoprotein-cholesterol; TG, triglycerides; VLDL-C, very low-density lipoprotein-cholesterol. ^*^, *p* < 0.05, compared to SO-enriched diet.

**Table 2:** Fold change, variance importance in projection score and receiver operating characteristics curve for the significant plasma metabolites after participants consumed the SO- and PHSO-enriched diets.

Metabolites	Fold		VIP Score ^2^	ROC Curve	
	Change ^1^	FDR		AUC ^3^	*p*-Value
TG 56:9	2.35	5.42 × 10^−4^	3.51	1.00	7.55 × 10^−6^
TG 54:8 (TG 18:2_18:3_18:3)	3.97	3.80 × 10^−4^	3.07	0.96	3.30 × 10^−4^
Benproperine	2.23	6.70 × 10^−4^	3.06	0.93	3.45 × 10^−4^
DG 36:5	2.01	6.20 × 10^−5^	2.76	0.90	1.97 × 10^−3^
TG 48:5	2.91	9.24 × 10^−3^	2.59	0.87	4.31 × 10^−3^
TG 54:7	2.92	7.52 × 10^−4^	2.93	0.87	7.93 × 10^−4^
TG 54:6 (TG 18:2_18:2_18:2)	2.03	6.05 × 10^−3^	2.40	0.78	9.57 × 10^−3^

^1^ The Benjamini & Hochberg procedure was conducted to account for multiple comparisons, and statistical significance was defined as FDR < 0.05; ^2^ The variable importance in projection score was calculated using partial least-squares discrimination analysis; ^3^ AUC-ROC curves were performed (cutoff: 0.7); DG, diglyceride; TG, triglyceride.

**Table 3:** Pearson Correlation Coefficient between plasma metabolites and VLDL, LDL and HDL lipoprotein fractions after subjects consumed the SO- and PHSO-enriched diets.

Plasma Metabolite	Diet	VLDL	LDL	HDL
TG 56:9	SO PHSO	0.639 * 0.286	0.267 0.793 *	−0.358 −0.074
TG 54:8 (TG 18:2_18:3_18:3)	SO PHSO	0.375 0.464	0.063 0.603	−0.087 −0.240
TG 48:5	SO PHSO	0.192 0.005	−0.444 −0.348	−0.509 −0.143
TG 54:7	SO PHSO	0.398 0.486	0.120 0.547	−0.080 −0.245
TG 54:6 (TG 18:2_18:2_18:2)	SO PHSO	0.379 0.410	−0.055 0.357	−0.345 −0.277
DG 36:5	SO PHSO	0.054 0.087	−0.090 0.255	−0.046 −0.049
Benproperine	SO PHSO	0.772 * 0.580	0.069 0.038	−0.065 −0.180

Values were presented as the correlation coefficient (*r*). *, *p* < 0.05. DG, diacylglycerol; HDL, high-density lipoprotein; LDL, low-density lipoprotein; TG, triglyceride; DG, diglyceride.

## Data Availability

Due to privacy and ethical restrictions, the data that support the findings of this study are available from the corresponding authors upon reasonable request.

## Supplementary Materials

The following supporting information can be downloaded at: https://www.mdpi.com/article/10.3390/metabo13040474/s1, Table S1: Composition of the two experimental diets; Table S2: The electrospray ionization mode, m/z value and retention time for the metabolites with Variable importance projection scores >1.7; Figure S1: Partial least squares discriminant analysis (PLS-DA) of the plasma metabolites in participants after they received soybean- or partially-hydrogenated soybean (PHSO)-enriched diets.

## Abbreviations

CMRFs: cardiometabolic risk factors; CHD, coronary heart disease; CVD, cardiovascular disease; FDR, false discovery rate; GCTOF MS, gas chromatography/time-of-flight mass spectrometry; HDL-C, high-density lipoprotein-cholesterol; iTFA, industrial *trans* fatty acid; LDL-C, low-density lipoprotein-cholesterol; PC, phosphatidylcholine; PE, phosphatidylethanolamine; PHSO, partially-hydrogenated soybean oil; PLS-DA, partial least squares discriminant analysis; ROC curve, receiver operating characteristics curve; rTFA, ruminant *trans* fatty acid; SO, soybean oil; TC, total cholesterol; TFA, *trans* fatty acid; TG, triglyceride; UHPLC-QTOF MS/MS, ultra-high pressure liquid chromatography/quadrupole time-of-flight tandem mass spectrometry; VIP, variable importance projection; VLDL-C, very low-density lipoprotein-cholesterol
