# Supplementary material for: Comparison of Plasma Metabolome Response to Diets Enriched in Soybean and Partially-Hydrogenated Soybean Oil in Moderately Hypercholesterolemic Adults-A Pilot Study
Source: Metabolites. 2023 Mar 26;13(4):474. doi: 10.3390/metabo13040474 (PMC10140885; doi:10.3390/metabo13040474)
Supplement: Supplementary file 1 [file metabolites-13-00474-s001.zip › metabolites-2230776-supplementary.pdf]

Supplementary Table S1. Composition of the 2 experimental diets

Variables	SO-enriched diet	PHSO-enriched diet
	% energy	
Carbohydrate	52.1	53.9
Protein	16.7	17.2
Total fat	31.2	28.9
SFA	6.52	7.25
MUFA	6.48	9.95
Trans 18:1	0.18	1.85
PUFA	12.7	8.66
Trans 18:2+18:3	0.43	0.67
Cholesterol (mg/1000kcal)	57	63
Fiber (g/1000 kcal)	13.6	13.4

The table was adapted from the parent study [24]. PHSO, partially-hydrogenated soybean oil; MUFA, monounsaturated fatty acids; PUFA, polyunsaturated fatty acids; SFA, saturated fatty acids; SO, soybean oil.

Supplementary Table S2. The the IUPAC International Chemical Identifier, electrospray ionization mode, m/z value and retention time for the metabolites with Variable importance projection scores > 1.7

Metabolites	VIP score	InChI ID	ESI mode	m/z; Quant m/z*	RT; RT index*
TG 56:9	3.51	HTODETRMAVZETG-GOCHBVGNSA-N	ESI (+)	918.7587	9.23
PE O-38:6(PE O-18:1_20:5)	3.46	KCNBSSYOJRUKOM-UHFFFAOYSA-N	ESI (-)	748.5292	6.02
SM d36:3	3.32	YMTVMVYOUDDTQJ-UOMMIRHQSA-N	ESI (+)	727.5731	4.34
PC 38:4(PC 18:1_20:3)	3.21	JNKAOGSDBBXHEP-UHFFFAOYSA-N	ESI (-)	868.6069	5.73
TG 54:8(TG 18:2_18:3_18:3)	3.07	IMPMCCKWZKDAGE-UHFFFAOYSA-N	ESI (+)	892.7405	8.97
Benproperine	3.06	JTUQXGZRVLWBCR-UHFFFAOYSA-N	ESI (+)	310.2109	1.59
PE P-40:7 or PE O-40:8	2.97	UKPNUUDYLNCIOE-IZHHWLPXSA-N	ESI (-)	772.5287	5.67
TG 58:10	2.94	GXWBCAVCOMAOHT-VMCJOIRWSA-N	ESI (+)	944.7714	9.42
TG 54:7	2.93	PRMRBZVOTCJWQD-UHFFFAOYSA-N	ESI (+)	894.7612	9.36
PC 36:3	2.84	BXRLDROZWDUSGM-ZRYFCQOPSA-N	ESI (+)	784.5864	5.14
PC 36:5	2.84	SUZYROYNFNQALJ-MHEIZRSESA-N	ESI (+)	780.5522	4.22
DG 36:5	2.76	PGXBELQFNRPKBC-WBVIKXMWSA-N	ESI (+)	632.5231	5.42
PC 36:1	2.76	ATHVAWFAEPLPPQ-VRDBWYNSSA-N	ESI (+)	788.615	6.09
TG 54:8	2.73	BMPVTDWOWBNPJU-NYRSPQLFSA-N	ESI (+)	892.7392	9.15
PC O-36:3	2.67	GDJAIASCRXEGBE-UHFFFAOYSA-N	ESI (+)	770.6038	5.50
PC 37:5	2.64	GGSPKQXXUZFLNX-UHFFFAOYSA-N	ESI (+)	794.5871	4.80
PC P-38:3 or PC O-38:4	2.63	GWBOVQHRCURSPU-QMFAPAEZSA-N	ESI (-)	854.6267	4.46
PC 36:5	2.60	DYDDZDMJSQYFGN-OIVUZXIWSA-N	ESI (-)	838.56	4.63
TG 48:5	2.59	-	ESI (+)	814.6918	8.71
PC 35:1	2.52	MFHIZGSSDZJFKD-IYEJTHTFSA-N	ESI (+)	774.598	5.77

PE P-38:6 or PE O-38:7	2.51	WVGALBKSWOUIEZ-XNHMFJFDSA-N	ESI (-)	746.5137	5.63
DG 36:4	2.47	MQGBAQLIFKSMEM-ZHARMHCNSA-N	ESI (+)	634.5413	5.88
PC O-36:4	2.46	AMIRMXUBGHUMRM-UHFFFAOYSA-Ns	ESI (+)	768.5922	5.41
PC O-44:6	2.45	IYYVNINVOLHLIG-UHFFFAOYSA-N	ESI (+)	876.6829	6.63
TG 54:6(TG 18:2_18:2_18:2)	2.40	HBOQXIRUPVQLKX-UHFFFAOYSA-N	ESI (+)	896.7751	9.75
PC 34:1	2.33	WTJKGGKOPKCXLL-VYOBOKEXSA-N	ESI (+)	760.5895	5.45
PC P-36:1 or PC O-36:2	2.31	ZOTYCHIFTCFAHC-KOUVQCMKSA-N	ESI (-)	830.6269	6.57
TG 56:8	2.27	UBGUHMDKBGQUND-VPFWBQFRSA-N	ESI (+)	920.7745	9.67
PC P-38:3 or PC O-38:4	2.16	GWBOVQHRCURSPU-QMFAPAEZSA-N	ESI (-)	854.6267	6.46
PC 33:1	2.14	OFRWLQSOUIZELQ-VEAYGOGPSA-N	ESI (+)	746.5717	5.14
TG 51:5	2.14	DYYKLAQWCNBXRJ-UNZGPCCPSA-N	ESI (+)	856.7463	9.55
TG 50:5	2.13	AFTBPUXZTDLRSP-UDQIKIEDSA-N	ESI (+)	842.7215	9.29
SM 46:6;3O	2.13	PSFAINLNJQRVRQ-UHFFFAOYSA-N	ESI (+)	877.6781	6.64
Serine	2.13	MTCFGRXMJLQNBG-REOHCLBHSA-N	-	204*	395020*
TG 52:6(TG 16:1_18:2_18:3)	2.09	NOOJQDXMGLPOOA-UHFFFAOYSA-N	ESI (+)	868.7461	9.31
TG 54:7(TG 16:1_18:2_20:4)	2.09	PRMRBZVOTCJWQD-UHFFFAOYSA-N	ESI (+)	894.7633	9.61
DG 38:6	2.08	YDVDXUYJFQLPEG-CTHJWPIASA-N	ESI (+)	658.5394	5.73
Glycerophosphocholine	2.07	SUHOQUVVVLNYQR-MRVPVSSYSA-N	ESI (+)	280.0919	8.34
PC P-34:1 or PC O-34:2	2.02	MBRHHFWRXQYYAN-JEPFLRBFSA-N	ESI (+)	744.5901	5.34
TG 58:9(TG 18:1_18:2_22:6)	1.98	RLAPDVQGJNAHOC-UHFFFAOYSA-N	ESI (+)	946.785	9.81
TG 54:2(TG 18:0_18:1_18:1)	1.97	RYNHWWNZNIGDAQ-UHFFFAOYSA-N	ESI (+)	904.8436	11.22
PC 32:2	1.94	GPWHCUUIQMGELX-VHQDNGOZSA-N	ESI (+)	730.538	4.40
PC 33:2	1.92	SBNDHGBVMZMSNL-UESLNCBNSA-N	ESI (+)	744.5529	4.68

PC P-40:5 or PC O-40:6	1.92	KUHMJRMPHBRAMY-DDURNVNNSA-N	ESI (+)	820.6213	5.69
2,3-dihydroxybutanoic acid	1.88	LOUGYXZSURQALL-UHFFFAOYSA-N	-	292*	384796*
PC P-38:5 or PC O-38:6	1.87	ATTCDOPAYPGSLE-LQULQHAGSA-N	ESI (-)	850.5948	5.58
PC 40:7	1.86	BPUROMFCPFGBOT-ZEGPSQTJSA-N	ESI (-)	890.5906	5.26
PI 36:1	1.85	YOBFISPJJWPPTK-JUCSSFOXSA-N	ESI (-)	863.5665	5.41
Linolenic acid	1.80	DTOSIQBPPRVQHS-PDBXOOCHSA-N	ESI (-)	277.2182	1.67
TG 54:3(TG 18:0_18:1_18:2)	1.79	CNGSOZKJBWHOEG-UHFFFAOYSA-N	ESI (+)	902.8325	10.86
TG 54:8(TG 14:0_18:2_22:6)	1.79	AINACBPMKQPREK-UHFFFAOYSA-N	ESI (+)	892.7395	9.28
Tetradecylsulfate	1.73	URLJMZWTXZTZRR-UHFFFAOYSA-N	ESI (-)	293.1784	1.10
PC O-34:2	1.72	WBAZRZTVRJLENP-UHFFFAOYSA-N	ESI (+)	744.5881	5.75

Asterisk indicates the data were from gas chromatography time-of-flight mass spectrometry. DG, diacylglycerol; ESI, electrospray ionization; InChI, the IUPAC International Chemical Identifier; PC, phosphatidylcholine; PE, Phosphatidylethanolamine; RT, retention time; SM, Sphingomyelin; TG, triacylglycerol.

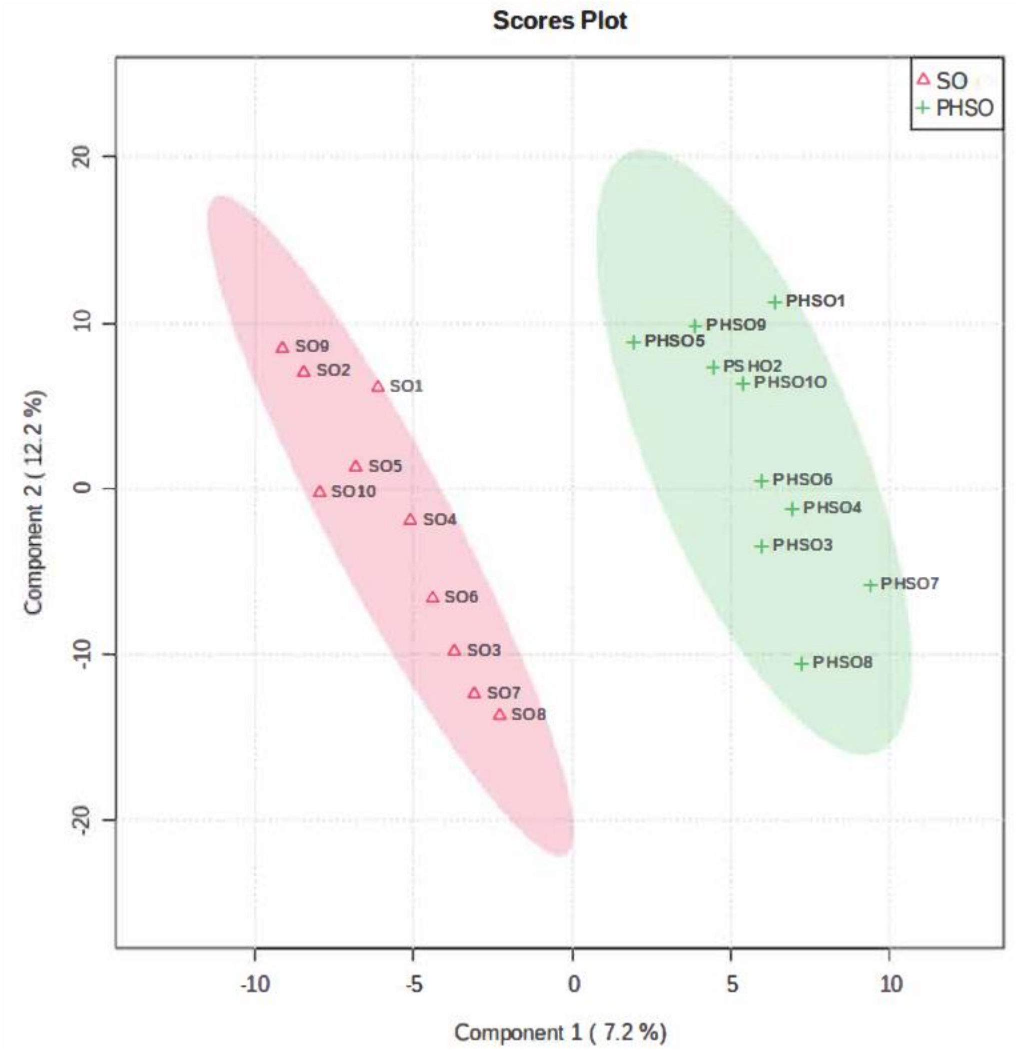

Supplementary Figure S1. Partial least squares discriminant analysis (PLS-DA) of the plasma metabolites in participants after they received soybean- or partially hydrogenated soybean (PHSO)-enriched diets
